# Possibilities and Expectations for mHealth in the Pacific Islands: Insights From Key Informants

**DOI:** 10.2196/mhealth.4626

**Published:** 2016-01-20

**Authors:** Elaine Umali, Judith McCool, Robyn Whittaker

**Affiliations:** ^1^ Epidemiology and Biostatistics School of Population Health University of Auckland Auckland New Zealand; ^2^ National Institute for Health Innovation University of Auckland Auckland New Zealand

**Keywords:** mHealth, Pacific Islands, prevention, health systems, health policy

## Abstract

**Background:**

The increase in mobile phone use across the globe is creating mounting interest for its application in addressing health system constraints. Although still limited, there is growing evidence of success in using mobile phones for health (mHealth) in low- and middle- income countries. The promise of mHealth to address key health system issues presents a huge potential for the Pacific Island countries where mobile use has radically increased. Current projections indicate an improved information and communications technology (ICT) environment to support greater access to mobile and digital devices in the Pacific region.

**Objective:**

The objective of the study was to explore key stakeholder perspectives on the potential for mHealth in the Pacific region.

**Methods:**

A series of in-depth interviews were conducted either face-to-face, via Skype or by email, with a series of key informants from the Pacific Rim region. Interviews were audio-recorded and later transcribed for detailed thematic analysis.

**Results:**

We found widespread support for the potential to use mobile phones as a mechanism to facilitate improved health service delivery in the region. Essential elements for the successful development and implementation of mHealth were identified by these stakeholders. These included: developing an understanding of the local context and the problems that may be usefully addressed by the addition of mHealth to existing strategies and services; consideration of local infrastructure, capability, policy, mobile literacy and engagement; learning from others, particularly other low- and middle-income countries (LMICs); the importance of building supportive environments and of evaluation to provide evidence of impact and total cost.

**Conclusions:**

The rapid growth of mobile phone use in the region presents a unique juxtaposition of opportunity and promise. Though the region lags behind other LMICs in the adoption of mHealth technologies, this offers the convenience of learning from past mHealth interventions and applying these learnings to achieve scale, sustainability and success. This study deepens the understanding of the potential of mHealth for the region, and offers a baseline from which discussions can be made to examine the limitations, barriers and complexities inherent in mHealth applications.

## Introduction

The rapid spread of mobile technology (mobile phones) has become the impetus for the recognition of its potential to create economic opportunities and enhance developmental interventions through mobile tools [1-2]. The traction of “mobile health” or “mHealth” within the health sector relies on its potential to extend the reach of health information and services, particularly among vulnerable populations [3].

Conservatively, it is estimated that almost 60 per cent the Pacific region population have access to a mobile phone [4]. According to the Groupe Speciale Mobile Association (GSMA) the Asia Pacific region will increase mobile subscribers from 1.7 billion in 2013 to 2.4 billion by 2020 [5]. Of course, the Asia Pacific region includes some of the world’s wealthiest and poorest countries, as well as the largest population. However, despite massive demographic and economic differences across the region, one factor remains; mobile phone use is expanding, rapidly [5]. As of 2015, a series of major infrastructure operations are underway to enhance the coordination and delivery of information technology services across the region [6]. The establishment of the Pacific Regional Infrastructure Facility (PRIF) has indicated a concerted effort to ensure donor partners in the region work together to maximize efforts to improve information and communications technology (ICT) infrastructure.

mHealth remains a novel practice in the Pacific region, a situation that brings with it some considerable opportunities. Despite increased access to mobile phones and network coverage in the Pacific Islands, mHealth has remained small-scale, restricted to pilot projects, and not integrated into the mainstream public health systems [7]. The challenges of initiation reflect a lack of local technological knowledge and, therefore, empirical evidence of efficacy of mHealth specific to the region. Moreover, the extent to which mobile literacy has kept pace with the expansion of Pacific markers is not clear [3,8]. To understand more about expectations, opportunities and challenges for building mHealth in the Pacific region, we sought the perspectives of a range of key stakeholders.

In this paper we present formative research findings on stakeholders’ views on the potential of mobile phones to improve health service delivery in the Pacific region. The experiences of health workers in the Pacific region and mHealth innovators in other low and middle-income countries, offer valuable insight into how policy makers and program developers can capitalize on previous experiences on mHealth interventions to ensure benefits are experienced in the Pacific region. The aim of the study was to explore key stakeholder perspectives on the potential for mHealth in the Pacific region, and how it could fit into existing broader health system structure.

## Methods

### Sampling

As this study was concerned with exploring the potential of mHealth in the Pacific region, a purposive sampling strategy was used to identify key informants that could provide a rich and diverse interview data. An initial environmental scan was conducted to identify possible key informants. Participants were selected for their expert knowledge and experience of Pacific life and health status. The key informants included participants from organizations that could potentially become stakeholders for mHealth initiatives in the region as well as professionals with regional or in-country experience implementing public health initiatives.

However, as the study progressed and new categories emerged from preliminary data analysis, it became obvious that more sampling was needed to develop these categories further. Chenitz and Swanson [9] described this aspect of theoretical sampling as the need to collect more data to “examine categories and their relationships and to assure that representativeness in the category exists”. As there was no precise number of predetermined key informants, additional sampling was added until preliminary data analysis showed that concepts have achieved theoretical saturation. The additional key informants were recommendations from participants and mobile solutions expert with a proficient understanding of mHealth initiatives in low- and middle-income countries.

### Data Collection

All interviews were conducted by one of the investigators (EU). In order to facilitate access to informants, including those who were based in different regions of the world, interviews were done in person, or online. Prior to the interviews, an introductory email and information sheet were provided to all potential participants. The participants who indicated interest to participate in the study were sent copies of the consent form.

The online interviews were either synchronous or asynchronous [10]. The synchronous interviews used online video call applications (ie, Skype or Google Hangouts). Whenever possible, face-to-face interviews and online video calls were preferred, however due to poor Internet connection especially in some Pacific countries, some interviews were conducted via Email. This form of interview has been one of the most popular Internet mediated methodologies to date [10].

The interview schedule was developed after an initial review of literature. The interview process was semi-structured and open-ended to allow the participants to focus and expound on issues they deemed important. The interviews followed a series of questions developed after an initial review of literature. Although the questions had specific themes, some topics were allowed to emerge during the course of the interviews. The synchronous online interviews and face-to-face interviews started with a short introduction, an explanation of the research, and a brief description of how mHealth is defined for the purpose of this research. The asynchronous interviews consisted of an introductory Email and a brief discussion on how to answer the interview questions that came as a separate attachment. The participants were given two to three weeks to return completed questionnaires. Additional follow-up or clarificatory questions were sent to key informants when necessary. All face-to-face interviews and online video calls ranged between 30 to 50 minutes.

The ethics approval for this study was granted by the Ethics Committee of the University of Auckland.

### Data Analysis

Interviews were digitally recorded and transcribed by the lead researcher. A preliminary data analysis was conducted during data collection to ascertain if there is a need to collect more data to examine emerging categories, their relationships, and to assure their validity in relation to other emerging themes [9]. This involved the analysis of interview transcripts after each interview to identify gaps and issues that needed to be clarified in subsequent interviews.

The coding and succeeding data analysis involved an open-coding process, where verbatim transcripts were coded, then re-coded according to an agreed coding framework that reflected the dominant themes within the text. Joint coding and analysis were conducted with other investigators to increase validity and reliability of the coding process.

## Results

A total of 19 key informants participated in the interviews. In the final sample, 8 were health professionals representing 8 Pacific island countries, 6 held regional public health roles in the Pacific region, 3 of which have some experience with mHealth, and 5 were from organizations in Africa, the United States, and Asia, widely involved in mHealth in developed and developing countries in areas such as research, planning, implementation, evaluation, policy-making and advocacy.

The analysis of the respondents’ interviews led to the identification of several themes which can be classified into nine key considerations for the development and scale-up of mHealth in the Pacific region. Verbatim quotes are present to illustrate and support the analyses. Quotes are presented by reference to their contributor (Pacific Health Provider, Regional Health Representative, and mHealth Practitioner). A brief background on the current mHealth activities in the region as discussed by key informants is also provided.

### mHealth Activities in the Pacific

All participants from each of the Pacific island countries (n=8) and regional public health representatives (n=6) knew of some form of mHealth activity in the region although they were not well aware of how these initiatives were implemented. Most participants had heard of the initiatives as second hand information—from colleagues or conference presentations—but most of these had been small-scale activities or pilot projects that were not part of any large-scale health program. As one interviewee expressed,

I am aware of mHealth activities but I don’t think it’s been clearly thought-through program or approach to improving health.

Regional Health Representative 4

The earliest mHealth activity mentioned was an outbreak detection system in Fiji using mobile phones to report cases such as diarrhea, measles, dengue, prolonged fever and rash observed in outpatient clinics. This was initiated in 2008 and used basic or feature phones programmed to have an additional menu that could send data directly to an online database.

### Start With the Problem—Then Ask If mHealth Can Contribute

Participants were adamant that the need for mHealth in the region was based on what the current health programs provided and whether there were gaps in health delivery. This was widely considered the first step to be taken before even considering using mobile technology. Identifying these problems can be done through a thorough review of current strategies and consultations with local and national health authorities.

We basically start with a problem first. What is the problem? Lost to follow-up? Referrals aren’t happening? Information gaps? Lack of communication between health workers? When we hear about those kinds of problems then we start thinking, okay, there might be a way to apply mobile technology or just eHealth technology to this.

mHealth Practitioner 2

Identifying problems in the health system also means identifying particular groups who are affected by these problems. Health problems affecting large groups of people were considered by participants to be more easily identifiable because of the significant burden they impose on the region’s health resources. In essence, the participants agree to focus on the existing health system challenges before considering whether mHealth, if at all, could provide solutions.

...we have to identify the gaps that mHealth could fill on our needs. We have to look at the gaps and the capacity of mHealth whether mHealth alone can fill those gaps. We have to study how mHealth will interact with other things.

Pacific Health Provider 2

Despite a lack of direct experience with mHealth, participants from the region concurred that using mobile technology may offer some advantages as well as limitations. Similarly, detailed knowledge about the health problems in-country forms the basis for decision-making about the value of introducing mHealth. This view was echoed by the mHealth practitioners who were swift to acknowledge the need to know each health system and current priorities before embarking on the journey into mHealth.

So part of the real question that you should ask is what are the major challenges to achieving better health outcomes in the Asia-Pacific region, and according to those challenges what are the reasonable mHealth strategies that have worked in other places that can help us...overcome these challenges that are particular to this region.

mHealth Practitioner 4

Being “problem-centered” moves the discussion away from mHealth to a more comprehensive approach to solving the challenges that are preventing the region from achieving better health outcomes. For some participants, the concept of looking at mHealth as the best solution potentially overshadows alternative system strengthening or policy-related intervention.

...look at how mobile fit into existing health system. Do we need mobile? Is there a different solution? So it’s very easy to think straight from... especially like me, I’m someone who always thinks from the mobile perspective. That’s what you first need to see—what is the challenge?

Regional Health Representative 6

### Tailor Interventions to the Local Environment and Local Needs

Since mHealth is largely technology dependent, participants emphasized the need to look at the capacity of current mobile technology in each country; this includes the mobile network coverage of the country, as well as the quality of mobile connectivity. Although the mobile infrastructure in the Pacific Islands region is gaining momentum in recent years, participants were quick to note that there are still areas that do not have mobile connectivity. One participant noted that

when you talk about Kiribati, if I remember correctly, it has about 60 islands, but mobile phone coverage is only in Tarawa and probably Christmas Island...

Pacific Health Provider 1

The recent deregulation of the telecommunications industry in some countries of the Pacific was thought to have helped improve mobile networks in many remote areas and significantly reduce the cost of mobile subscriptions. Participants believed that similar policy attempts in other Pacific countries to introduce competition and improvements in service could help improve access to mobile networks in remote areas of the region. One commented that

I think the policy attempts to introduce competition in the telecoms market would probably open up possibilities.

Regional Health Representative 3

Knowing the capacity of mobile connections in the area to identify which types of mHealth applications would best suit certain areas was considered a core priority when considering mHealth in the region. Participants also mentioned the need to look at existing policies that could affect mobile technology use and identify the gaps in these policies so that standards can be put in place to support mHealth initiatives. One interviewee observed the need to enable,

...environment for the use of technology—whether it’s looking at policies, the frameworks, the standards that need to be put in place as well as the human resource needs over the short and long term...

mHealth Practitioner 4

The existing health infrastructure is quite an essential part of the enabling environment. If the current public health system is largely dysfunctional at supporting existing infrastructure, the introduction of a new innovation such as mHealth would most likely be ineffective.

...look at the capacity of the system you are building on top of. What kind of capacity is in place to support existing health structures to deliver information, respond to new influx of patients who maybe responding to information they received? Is the system in place to support those people? Start not necessarily looking on the solution, but on the support structure that exist to support the solution.

mHealth Practitioner 2

In addition to understanding the technology environments, participants mentioned the importance of generating a detailed understanding of the users of mobile technology. One participant (mHealth practitioner 6) emphasized the need to invest in ethnographic research prior to starting anything else. Some essential questions to answer include: who is using mobile phones—is it the women or the men, younger generation or older—and who controls the device? In the Pacific Island region, cultural and societal barriers could also affect phone ownership and use. Women in some societies need to ask for permission from a man before using or owning a mobile phone.

...fathers or husbands don’t necessarily want their wives to own a mobile phone. They say it will be a means for them to go rogue, to have boyfriends, have an affair. And then women don’t necessarily want a mobile phone if they think their families can use it to control them.

mHealth Practitioner 3

Mobile phones are often shared among members of the family, creating potential barriers (eg, for sexual health) and opportunities (eg, for healthy eating) for different mHealth initiatives. Participants also noted that there might be generational gaps in phone usage or preferences in certain mobile phone functions (ie, SMS or voice calls) among the population.

I think finding out who has coverage, who has mobile phone, who in the family—is it just the father who has a mobile phone, or the whole family.

Pacific Health Provider 5

I think it’s really important again to go back to like the ethnography piece and really understand who they are or how they interact with the world, how they interact with technology, how they communicate, how they access services already, and then delve that into the design of technology solutions.

mHealth Practitioner 5

A few respondents raised the issue of the perceived integrity of research in the Pacific. However, the majority of participants valued the contribution of dedicated research to understand the nuances of potential users, particularly when the intervention aims to promote behavior change.

Our instinct is to rush to build solutions on top of mobile devices without really seeing if the population is ready to use those solutions.

mHealth Practitioner 2

...when you’re looking into behavioral, behavior-related programs that really understanding the users and the potential beneficiaries whatever technology solution or content you are planning to implement is really, really important.

mHealth Practitioner 5

### Can Lessons Be Learnt From Other Settings?

Most of the respondents suggested studying the lessons learned in other low- and middle- income countries that have already implemented mHealth. Identifying what worked in previous initiatives, and what didn’t work, can help in the decision-making and design of future activities.

I think the other is also to look into what other people are doing and see what range of activities should be explored in mHealth. And then think about it from that perspective as well—what’s applicable, which ones are aligned with what we are trying to achieve.

Regional Health Provider 5

A few respondents warned about “innovating in isolation”. They suggested of looking at what is being implemented in the region to see how it could complement or work with planned mHealth programs. Interviewees mentioned trying not to “reinvent the wheel” as it could cause duplication of efforts and fragmentation which can reduce the effectiveness of mHealth solutions. As one said,

Look at the evidence and look at what is working, what scaling, what lessons learned were garnered through other implementation so you don’t have to like, one, start from scratch, reinvent the wheel.

mHealth Practitioner 5

### Genuine Engagement From the Start

Key informants emphasized the importance of engaging potential stakeholders early on in the implementation of the mHealth strategy. Strong partnerships were considered imperative from the very beginning of any initiative planning stage. A participant mentioned that engaging people in the government to get their perspective in defining what the key challenges are is important when looking at establishing a broad mHealth strategy for the Pacific region.

I think it is important to engage governments from the outset, so particularly if you are looking at public health interventions. Ultimately it’s the government who has to sustain it. And so one of the biggest lessons learned is that a lot of programs fail because there is no government intervention. And it’s not even “get government engagement eventually”, it’s “get government engagement from the beginning”

mHealth Practitioner 5

Strategic partnerships with the private sector, especially the mobile providers, are also crucial. Mobile network operators are well placed to provide technical expertise, resources and network to help operationalize and scale up the project. According to participants, the private sector involvement is crucial for any mHealth strategy but the public sector needs to set the priorities and direction, as the ultimate driver of the mHealth strategy.

...private sector partnerships are really important, a lot of these things are things that the private sector is well-placed. But it really needs to be defined by the public sector and the priorities need to be set by the public sector and the guidance need to be set by the public sector. But a lot of the types of like applications and tools that need to be implemented could easily be done by the private sector.

mHealth Practitioner 5

Participants also highlighted end-user engagement. Having health-providers and community involved, as they are the ultimate end-user of mHealth, is important in every phase of the mHealth strategy. One participant mentioned that a current trend in many developing countries right now is to actively involve the civil society in providing feedback on what’s happening in their local communities. Encouraging social accountability and valuing end-user engagement is making an impact on how decisions are being made.

...we are starting to see people become more engaged and even the most vulnerable people are providing citizen feedback of what’s happening in their local communities. And I think that’s one major area where we’re already seeing some really good progress and inputs. And it’s making an impact on how the allocations are being made and that sort of thing which is really exciting.

mHealth Practitioner 5

Participants commented that mHealth initiatives will work best as a multisectoral approach. One of the main policy issues may include addressing how different stakeholders from the private and public sector, including NGOs and civil society, work together to mainstream mHealth as a viable solution to health problems.

cause you are dealing with unusual actors-you are dealing with telecommunications companies; you are dealing with phone companies-and these are people who are not used to working in the space of health. So it’s a new space for them and I think they need that guideline.

mHealth Practitioner 4

### Building a Supportive Health System

An issue that some participants raised was that implementing a mHealth program could be seen as an alternative to improving the health system infrastructure. These interviewees stressed that mHealth is a supplementary activity to existing health programs not a replacement. If core health infrastructures are defective or lacking, investment in mHealth cannot be used to replace investment to improve health services.

I see it [mHealth] more as a way to provide additional support for people without adequate services but it doesn’t make up for inadequate services...And certainly it should not be seen as an investment into the infrastructure of proper health services. It’s a supplementary activity, because the health services are inadequate at the moment and there’s insufficient capacity. So it should not be seen as supporting or encouraging investment not to be put into those services which are crucially needed.

Regional Health Representative 5

Adequate personnel or human resources to deliver quality health service must also be in place before the implementation of the mHealth initiative. As some mHealth applications create more demand for health services, the system must be ready to accommodate the influx of new patients or new users.

I would first look at how the health system works in general. Like for example, we can encourage people to go for a service, that’s what we do for mobile. But if there is no doctor available, if there are no medicines, it works counter effective.

mHealth Practitioner 1

### Building Local Capacity for mHealth

The majority of participants acknowledged the lack of technical capacity in mHealth as a challenge. mHealth will have a greater impact if stakeholders are trained and upskilled in the use of mobile technologies and users are educated to a higher level of mobile literacy. For instance, if the ultimate users of the mHealth tool are health workers, increased mobile literacy and understanding of ICT applications will save time and increase their efficiency, expressed succinctly by this interviewee

If you are collecting data using mobile phones, it will require training and specific skills set.

Pacific Health Provider 3

Participants suggested establishing a coordinating body composed of different stakeholders to establish standards and systematize efforts. This would allow for more coordinated and integrated efforts as well as sharing relevant data to aid decision-making.

...if there are 10 NGOs and they are all working with pregnant women, are they collecting at least the same few data elements that allow them to share information with the national health system or with each other? So it often helps to have some kind of national eHealth or mHealth committee or user-group or ministry agency which serves as the focal point for these activities within that country.

mHealth practitioner 4

Several participants advocated establishing a regional network that would endorse a regional approach to mHealth would be beneficial for the region for initial start-up or scaling-up. Having a regional network would encourage information sharing of best practices and innovative solutions that worked in other parts of the region.

### Designing Interventions With the End-User in Mind: Use the Best Technology Options

In order to maximize the potential of mHealth, the majority of participants recommended keeping the end-users’ wants and needs in mind when designing mHealth solutions. This underscores the earlier recommendation of doing an ethnographic study to understand the end-users. Data from earlier baseline studies as well as from the consultations with the end-users could feed into the design of the mobile application.

...go back to the end user time and time again. And by end user, I don’t mean the NGOs, I mean the person the NGOs are serving right down the end of the line.

Regional Health Representative 5

I don’t think there’s huge amount of research done around types of in terms of what people want, what beneficiaries want. And I don’t know if there’s research been done to what the health system feels will be useful.

Regional Health Representative 6

There is a range of available types of mHealth solutions. However, participants suggest that decision makers consider the level of mobile literacy of end-users and their response to the technology when choosing which mHealth solution to use. Simple mHealth applications with minimum technical requirements, for instance, would require less technical skills among end-users.

As the majority of the PNG [Papua New Guinea] population live (sic) in rural areas and as there is very low literacy levels, I believe simple mHealth applications that have the minimum technological requirements and require minimal effort and comprehension on the user’s behalf are best suited to PNG.

Pacific Health Provider 6

### Integrate mHealth Into Wider Program Strategies and Design for Scale

Participants commented that mobile solutions are not ‘stand-alone’ efforts, they serve as support mechanism to deliver better quality healthcare, and must align with national health system goals.

mHealth initiatives can’t be effective as ‘stand-alone’ efforts—there needs to be support and coordination with existing work that’s already taking place in the local communities.

Pacific Health Provider 9

...the mHealth strategy does not succeed in a vacuum, okay? That’s very, very critical to keep in mind. Any successful mHealth strategy has to be part of a multifaceted approach to solving a problem.

mHealth Practitioner 4

Mobile solutions can complement and support a wider program strategy. For example, in a behavior change communication initiative, mHealth is just one of the channels by which the health message could be delivered.

In designing the mHealth strategy, participants identified the need to plan for scalability. There have been quite a number of pilot projects but few examples of successful mHealth projects that have gone on to a national-level scale. The lack of foresight to plan for scale-up among pilot projects, limits the ability to be sustainable and the potential to deliver positive health outcomes. Administrators of mHealth initiatives must have the knowledge on how to scale-up pilot projects and integrate this knowledge from the outset.

One participant said,

we’re on a phase right now where we don’t need any more pilot projects.

mHealth Practitioner 5

The number of pilot projects in many low- and middle- income countries would provide extensive lessons on how important scalability is to the mHealth strategy.

### Evaluate the Whole Impact and Calculate the Real Cost

Cost is one of the biggest barriers to sustainability. Understanding the cost attached to mHealth can give a better perspective on how these costs could be managed. Participants identified two costs involved in the operation of mHealth: the cost to the individual of accessing the service, and the cost to the country of delivering the service.

Cost was widely perceived as an intractable challenge that users, especially from poorer countries, will face to access mHealth. How cost can affect the users’ willingness and ability to pay for using mHealth services should be carefully assessed, as this will affect the implementation and sustainability of the mHealth strategy. One participant wondered,

...if you want people to respond, does that mean people need to have money to top up their phones to respond or is it just messaging that’s going to be just for your information where they don’t need pay to respond to you?

Pacific Health Provider 5

Secondly, developing and implementing an mHealth strategy will incur costs. There will be fixed costs associated with the capital investments to set-up the technology required for the intervention, and operational and maintenance costs to sustain the system. One participant from the Pacific region expressed his skepticism about the financial viability of investing in mHealth. This is an opinion that might be shared by other Pacific countries with limited financial resources. One interviewee expressed it thus,

...we are very mindful of the operational cost. Investment cost can last for 12 months but the operational cost including the placement of the equipment is something that I don’t think the country can afford.

Pacific Health Provider 2

Most participants from the Pacific region are optimistic that telecommunication service providers could assume some of the cost for the mHealth strategy. They are banking on these companies’ commitment to social responsibility to provide their technical expertise and network connection. As one said,

I think it’s about selling the idea to governments, to the telecom and have their social responsibility be on board in that angle to reduce costs for sending texts

Pacific Health Provider 4

But for providers to come on board, participants highlighted the need to adopt business models that will provide mutual benefits for public-private partnerships. One participant argued that most people assume the companies stand to gain much financially from mHealth, although in reality this may be very limited. Therefore to leverage support from telecom companies, decision-makers need business models that will clearly define the opportunities and value that mHealth could deliver.

## Discussion

### Principal Findings

The acceleration of mobile connectivity across the Pacific, presents potential of mHealth to improve access to health information and quality of services across the region [11,12]. At face value, the concept of mHealth was widely considered an appealing option for supplementing and bolstering overworked or inefficient systems (surveillance, for example [13]). Similarly, the prospects for mHealth-based innovations in new domains such as noncommunicable disease (NCD) prevention, disaster response and maternal health, are well recognized [14]. However, overwhelmingly both our mHealth and local public health providers erred toward realism over optimism; the extent to which a mobile technology based initiative can remedy systemic issues was the challenge. The Pacific Islands region is facing some of the most significant health challenges ever to confront the region. Burgeoning NCD rates are overwhelming under-resourced health systems and infrastructure. A chronic lack of trained health and allied personnel places additional demands on donor-dependent countries. Yet, a cautious optimism was expressed among Pacific and international stakeholders.

At the recent Pacific Health Ministers Meeting in Fiji (April, 2015), a declaration by Dr Tuktuitonga of the Secretariat of the Pacific Community (SPC) speaks of the radical developments in the region and the need to draw upon the strengths and assets of all contributors—public and private for health gains:

The Pacific today is a different place than it was 20 years ago, and our region faces a multitude of challenges. We have an opportunity to build on the progress already achieved in Pacific health through increased cooperation between governments, non-governmental organizations, civil society and the private sector, to work together to improve the lives of all Pacific people [14]

14

The evidence is clear; the Pacific regional health challenges can only be overcome with coordinated, innovative and multisectoral actions led by the countries. Can mHealth play a part in these solutions? Time will tell. However, the foundations are firming; with young, increasingly media savvy populations, enthusiastic telecommunications sector and not yet saturated mHealth environment, timing is right to explore the possibilities.

### Limitations

A limitation of this study is that we interviewed people who were considered and recommended as potential users or stakeholders of current or future mHealth initiatives. It is likely therefore that we missed the opportunity to interview those who held contrary or unique perspectives that were not raised by our sample. Qualitative research methodologies, in general values subjectivity and accordingly there is potential for variation in interpretation of participants perspectives [15]. We have been fastidious in our coding and analysis process and used quotations to support our findings. Interviews conducted via Skype may well be qualitatively different than those conducted face to face. However, with consideration of limited resources and increasing technological capacity for Internet based interviews, this proved effective for our study. There were also numerous attempts to obtain a representative from the telecom industries present in the Pacific region, but none agreed to participate in the interviewed. Knowing what the telecom industries think about mHealth especially since they are essential stakeholders in mHealth implementation could have provided critical inputs to this study. At present, although deregulation and reforms in the telecommunications sector in the Pacific have driven the increase in mobile phone access in the region [4] it is largely unknown how receptive the telecom industries are to establish a presence through mHealth.

### Conclusions

This study deepens the understanding of the potential of mHealth for the region, and offers a baseline from which discussions can be made to examine the limitations, barriers and complexities inherent in mHealth applications. The experiences of developed and developing countries in implementing mHealth over the past years combined with local conditions of the region has revealed potential barriers and risks such as access to mobile phones, literacy, lack of complementary infrastructure and supportive environment, over-expectation and the underlying technical limitations of local institutions to implement mHealth. Although this research was done under the context of how mHealth could be adapted in the Pacific region, the findings of this research are also applicable and useful to other settings. What this study has established is to emphasize the primary importance of user-engagement, stakeholder collaboration and the careful consideration of local contexts to support long-term implementation of mHealth. These considerations cut across various mHealth applications and disease preventions initiatives in many countries. The biggest challenge for the Pacific region and in many countries is how to bring initial pilots to scale and become mainstreamed to national health system structures.

Finally, although the Pacific region lags behind other low- and middle- income countries in the adoption of mHealth technologies [8], this position offers the convenience of learning from past mHealth interventions and applying these learnings to adapt tools, achieve scale, sustainable positive impacts [3,16-18]. Cautious optimism is, however, the safest position as there is plenty of work still to be done to fully appreciate how to adapt this technology to achieve equitable beneficial outcomes within the Pacific Islands.

## Abbreviations

GSMA: Groupe Speciale Mobile Association
ICT: information and communications technology
NCD: noncommunicable disease
PNG: Papua New Guinea
PRIF: Pacific Regional Infrastructure Facility
SPC: Secretariat of the Pacific Community
WHO: World Health Organization
